# Glutamate-induced apoptosis in neuronal cells is mediated via caspase-dependent and independent mechanisms involving calpain and caspase-3 proteases as well as apoptosis inducing factor (AIF) and this process is inhibited by equine estrogens

**DOI:** 10.1186/1471-2202-7-49

**Published:** 2006-06-15

**Authors:** YueMei Zhang, Bhagu R Bhavnani

**Affiliations:** 1 Department of Obstetrics and Gynecology, University of Toronto, Institute of Medical Sciences, University of Toronto, Department of Obstetrics and Gynecology, St. Michael's Hospital, Toronto, Ontario, Canada

## Abstract

**Background:**

Glutamate, a major excitatory amino acid neurotransmitter, causes apoptotic neuronal cell death at high concentrations. Our previous studies have shown that depending on the neuronal cell type, glutamate-induced apoptotic cell death was associated with regulation of genes such as Bcl-2, Bax, and/or caspase-3 and mitochondrial cytochrome c. To further delineate the intracellular mechanisms, we have investigated the role of calpain, an important calcium-dependent protease thought to be involved in apoptosis along with mitochondrial apoptosis inducing factor (AIF) and caspase-3 in primary cortical cells and a mouse hippocampal cell line HT22.

**Results:**

Glutamate-induced apoptotic cell death in neuronal cells was associated with characteristic DNA fragmentation, morphological changes, activation of calpain and caspase-3 as well as the upregulation and/or translocation of AIF from mitochondria into cytosol and nuclei. Our results reveal that primary cortical cells and HT22 cells display different patterns of regulation of these genes/proteins. In primary cortical cells, glutamate induces activation of calpain, caspase-3 and translocation of AIF from mitochondria to cytosol and nuclei. In contrast, in HT22 cells, only the activation of calpain and upregulation and translocation of AIF occurred. In both cell types, these processes were inhibited/reversed by 17β-estradiol and Δ^8^,17β-estradiol with the latter being more potent.

**Conclusion:**

Depending upon the neuronal cell type, at least two mechanisms are involved in glutamate-induced apoptosis: a caspase-3-dependent pathway and a caspase-independent pathway involving calpain and AIF. Since HT22 cells lack caspase-3, glutamate-induced apoptosis is mediated via the caspase-independent pathway in this cell line. Kinetics of this apoptotic pathway further indicate that calpain rather than caspase-3, plays a critical role in the glutamate-induced apoptosis. Our studies further indicate that glutamate- induced changes of these proteins can be inhibited by estrogens, with Δ^8^,17β-estradiol, a novel equine estrogen being more potent than 17β-estradiol. To our knowledge, this is the first demonstration that glutamate-induced apoptosis involves regulation of multiple apoptotic effectors that can be inhibited by estrogens. Whether these observations can help in the development of novel therapeutic approaches for the prevention of neurodegenerative diseases with estrogens and calpain inhibitors remains to be investigated.

## Background

High concentrations (mM) of the excitatory neurotransmitter glutamate can accumulate in the brain and are thought to be involved in the etiology of a number of neurodegenerative disorders including Alzheimer's disease [1,2]. A number of invitro studies indicate that at high concentrations, glutamate is a potent neurotoxin capable of destroying neurons by apoptosis [3,4]. We and others have previously reported that glutamate induces characteristic oligonucleosomal DNA fragmentation (DNA ladder) and apoptotic cell death by up and down-regulation of Bax and Bcl-2, in a stable mouse hippocampal neuronal cell line HT22 which lacks caspase-3, the primary activator of apoptotic DNA fragmentation [5]. In contrast, in primary cortical cells, glutamate-induced cell death involves upregulation of caspase-3 and its activation via a caspase-dependent pathway involving mitochondrial signaling [6]. Glutamate-induced DNA fragmentation observed in HT22 cells implies that regulatory factors other than caspase-3 are involved in the apoptotic process in these cells.

Recent studies demonstrate that calpain, a calcium-dependent protease, and apoptosis inducing factor (AIF) can play an important role in apoptotic cell death via a caspase-independent apoptotic pathway [7-11]. Glutamate toxicity appears to involve a rapid Ca^2+ ^influx into neurons and these high levels of intracellular Ca^2+ ^are cytotoxic [12,13]. Ca^2+ ^can activate several key enzymes, including nitric oxide synthase (NOS) and proteases such as calpains and can also result in mitochondrial dysfunction [12,14]. Furthermore, a reduction in mitochondrial transmembrane potential has been reported to accompany AIF release and early apoptosis [15,16]. AIF is a ubiquitously expressed flavoprotein with significant homology to bacterial oxidoreductases and has NADH oxidase activity [17]. Following induction of apoptosis, AIF translocates from the outer mitochondrial membrane to the cytosol and the nucleus, resulting in the induction of nuclear chromatin condensation and large molecular weight DNA fragmentation in a caspase-independent manner [18,19]. Proteases such as caspases, calpains and granzyme B [20-22], have been reported to play a critical role in mediating apoptosis, especially the key modulator caspase-3. Similarly, calpains have been implicated in apoptosis in response to hypoxia and irradiation exposure in neuronal and non-neuronal cells [23]. Calpain is a calcium-dependent papain-like neutral cysteine protease, which is widely distributed in neurons [23,24]. A number of subcellular targets have been identified as substrates for calpain cleavage, including spectrin, microtubules-associated protein (MAP), tau and neurofilaments, however, the precise physiological role of calpain remains obscure [24]. Activation of calpain is triggered by an elevation of cytoplasmic free Ca^2+ ^concentration which results in the cleavage of various proteins and culminates in cell death [25]. Activation of calpain is an early event in the onset of apoptosis in thymocytes therefore inhibitors of calpain can reduce this process of cell death [26,27]. Calpain is also implicated in neuronal cell death associated with cerebral ischemia and other neuronal insults [28,29].

A number of studies have demonstrated that estrogens are potent antioxidants that can inhibit some of the neurotoxic effects of oxidative stress [4,30,31]. We and others have reported that estrogens can increase cell survival and attenuate *invitro*cell death induced by potential Alzheimer's disease-related insults, such as exposure to oxidized LDL and glutamate in a hippocampal cell line and primary cortical neurons [32-34]. The mechanism underlying neuroprotection by estrogens against glutamate oxidized neurotoxicity is not fully understood. Recently we have reported that neuroprotection of estrogens against glutamate cytotoxicity is associated with modulation of the expression of Bcl-2 family of genes and caspase-3 proteins, inhibition of cytochrome c release from mitochondria into the cytosol and prevention of DNA fragmentation [5,6]. In the present study, we selected two cell types, primary cortical cells and hippocampal cell line HT22, to investigate the roles of calpain, apoptosis inducing factor (AIF) and caspase-3 in glutamate-induced apoptosis and its prevention by estrogens. The estrogens chosen for this study were 17β-estradiol (17β-E_2_) and Δ^8^, 17β-estradiol (Δ^8^, 17β-E_2_), the latter being the more potent antioxidant [35,36].

## Results

### Expression of ERα and ERβ mRNA in rat cortical cells

**Figure 1:**
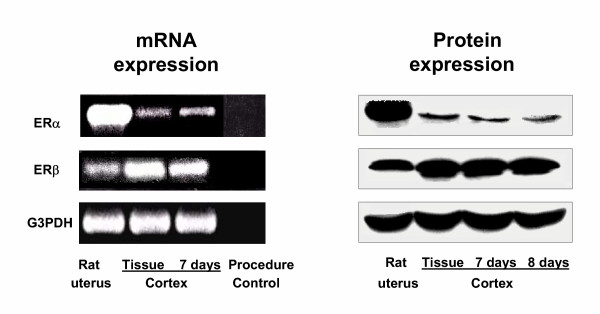
**Expression of estrogen receptors (ERα and ERβ) mRNA and protein in rat neuronal cortical cells**. Rat cortical cells were cultured for 7–8 days and total RNA and protein were extracted. RT-PCR and Western blot analysis were carried out as described under "Methods". Rat uterus was used as a positive control.

It has been reported that the HT22 cell line lacks estrogen receptors [4]. In order to ascertain expression and relative distribution of estrogen receptors ERα or ERβ in rat cortical cells, we performed RT-PCR and Western blot analysis (Figure 1). The results revealed that freshly isolated neuronal cortical cells or cells after 7–8 days in culture expressed both ERα and ERβ with the expression of ERβ being higher than that of ERα. Both subtypes of ER mRNAs and the corresponding proteins were found in the adult rat uterus (positive control), with ERα levels higher than those of ERβ. This is in keeping with a previous report [41].

### Anti-apoptotic effects of protease inhibitors of calpain and caspase-3 in mouse hippocampal cell line (HT22) and primary cortical cells

We have previously reported that glutamate treatment induces apoptotic cell death in HT22 cells and primary cortical cells in a time and dose-dependent manner [5,6]. We had shown that the commitment point to this death was ~6 hours after initiation of 1 mM glutamate exposure in primary cortical cells and for ~8 hour exposure with 7–8 mM glutamate in HT22 cells. Based on these preliminary data, all subsequent experiments were carried out with a 6 hour treatment with 1 mM glutamate in primary cortical cells and 8 hours with 7–8 mM glutamate in HT22 cells.

### Effects of calpain and caspase inhibitors on glutamate-induced cell death

#### (a) In mouse hippocampal cell line

**Figure 2:**
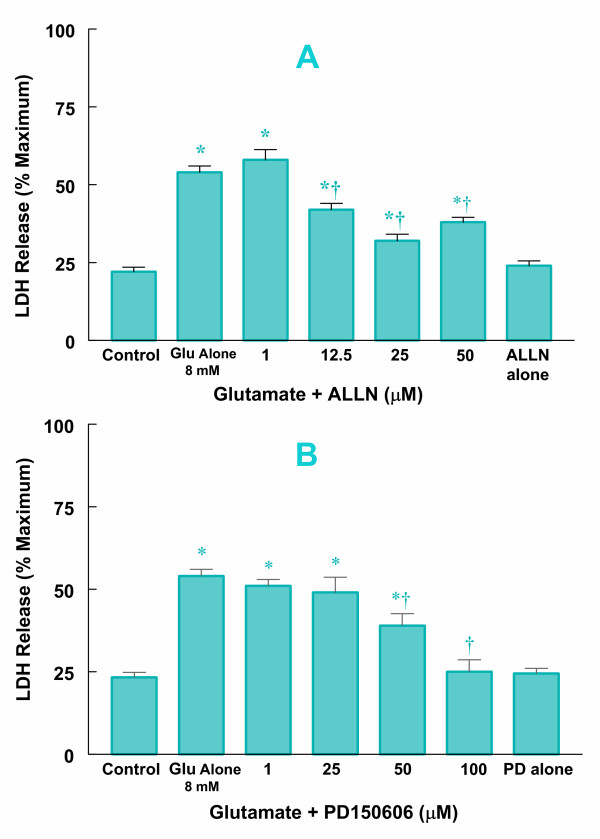
**Effect of various concentrations of calpain inhibitors, ALLN and PD150606, on glutamate-induced cell death in HT22 cells. **HT22 cells were treated with 8 mM glutamate in the presence or absence of 1–50 μM ALLN (A) and 1–100 μM PD150606 (B). Cell death was assessed by measuring LDH release after 8 hrs of treatment *P < 0.05 compared to control; **†***P *< 0.05 compared to glutamate alone.

**Figure 3:**
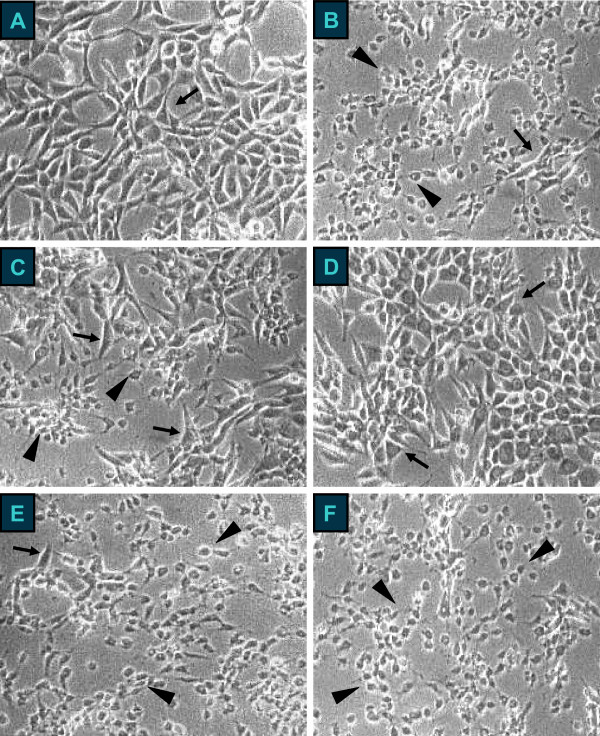
**Effect of calpain and caspase inhibitors on glutamate toxicity in HT22 cells as assessed by phase contrast microscopy (20×)**. HT22 cells were treated with 8 mM glutamate in the presence or absence of calpain inhibitors, 25 μM ALLN and 100 μM PD150606, and caspase inhibitors, 100 μM z-DEVD and 100 μM z-VAD, for 8 hrs. Normal cells are depicted by arrows and degenerated cells by arrowheads. A = untreated control cells; B = glutamate alone; C = glutamate + ALLN; D = glutamate + PD150606; E = glutamate + z-DEVD; F = glutamate + z-VAD.

Treatment of HT22 cells with 8 mM glutamate was initiated in the presence or absence of various concentrations of calpain I inhibitor (ALLN) or PD150606 for up to 8 hours. The results are depicted in Figures 2A and 2B. Glutamate (8 mM) alone caused a significant increase (50%) in cell death compared to control, while calpain inhibitors, either ALLN or PD150606, reduced glutamate-induced cell death in a dose-dependent manner. ALLN (12.5–50 μM) reduced cell death by 50–70% (Figure 2A). Similarly, 50 to 100 μM of PD150606 decreased cell death by 70–100% (Figure 2B). However, when cells were treated at a lower glutamate concentration (3–4 mM) for a longer period (16 hours) however, cell death increased by 40 to 50%, and this cell death was prevented by 12.5–50 μM ALLN (data not shown). In the absence of glutamate, neither inhibitor had an effect on cell viability. The effect of calpain inhibitors on glutamate-induced apoptotic cell death was also associated with characteristic morphological changes (Figure 3). After an 8 hour incubation with 8 mM glutamate, degenerated and dead cells were clearly visible in the culture (Figure 3B); the cellular extensions (dendrites) seen in untreated HT22 cells (Figure 3A) were retracted and the cells appeared rounded and had detached. In the presence of calpain inhibitors, 25 μM ALLN or 100 μM PD150606, most cells survived and had the appearance of normal untreated cells (Figure 3C and 3D). Only a small number of dead cells were visible in the presence of 25 μM ALLN (Figure 3C). In contrast, caspase inhibitors, either the specific caspase-3 inhibitor z-DEVD (Figure 3E) or a pancaspase inhibitor z-VAD (Figure 3F) at a concentration of 100 μM, failed to inhibit cell death induced by 8 mM glutamate. This cell death was further confirmed by using LDH release assay (data not shown).

#### (b) In primary cultures of rat cortical neuronal cells

**Figure 4:**
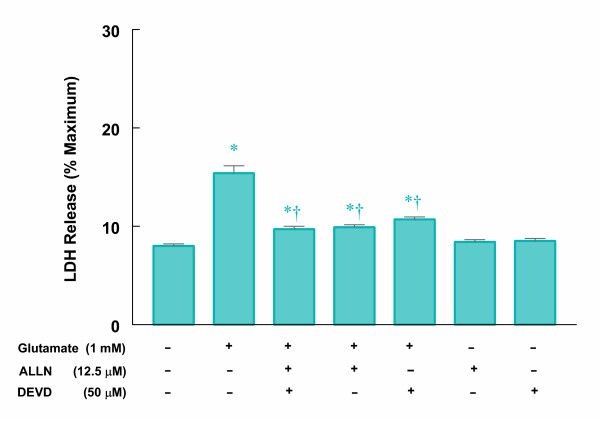
**Effect of calpain and caspase inhibitors on glutamate-induced cell death in primary cortical cells**. Primary cortical cells isolated from 18 day old fetal rat brains were cultured for 7–8 days and treated with 1 mM glutamate alone or in the presence of the optimal concentration of 12.5 μM calpain inhibitor ALLN and 50 μM z-DEVD caspase-3 inhibitor. LDH release (% maximum) was measured after 6 hrs of treatment *P < 0.05 compared to control; **†***P *< 0.05 compared to glutamate alone.

**Figure 5:**
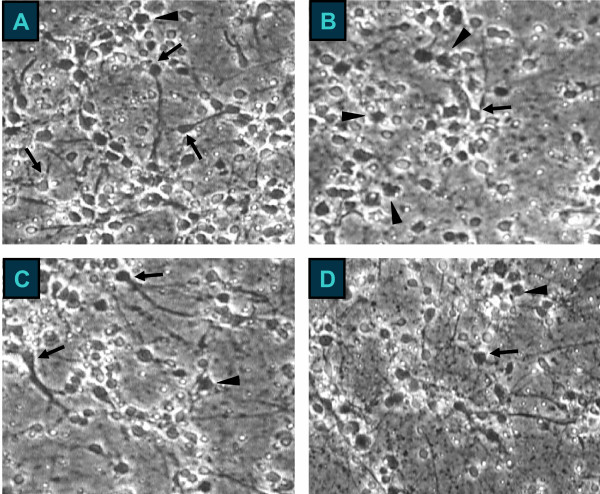
**Effect of calpain and caspase inhibitors on glutamate-induced cell death in primary cortical cells as assessed by phase contrast microscopy (20×)**. Primary cortical cells were isolated and treated as described under Figure 4. Normal cells are depicted by arrows and degenerated cells by arrowheads. A = untreated control cells; B = glutamate lone; C = glutamate + ALLN; D = glutamate + z-DEVD.

To determine the role of calpain and caspase-3 in primary cortical cells, cells were treated with 1 mM glutamate in the presence or absence of calpain inhibitor (12.5 μM ALLN) or specific caspase-3 inhibitor (50 μM z-DEVD) for 6 hours (Figure 4). Glutamate treatment alone induced one fold increase (50%) in LDH release or cell death compared to control. Caspase-3 inhibitor z-DEVD significantly inhibited glutamate-induced cell death by 60%. Similarly, the presence of calpain inhibitor ALLN resulted in a 70% reduction in (1 mM) glutamate -induced cell death. These data indicate that both caspase and calpain-mediated apoptosis were involved in glutamate-induced cell death in primary cortical cells. However, the addition of a combination of two protease inhibitors did not result in a greater decrease in LDH release or cell death, suggesting that calpain and caspase inhibitors has no synergetic effects on glutamate-induced cell death. Protection by both inhibitors was also detectable by characteristic morphological changes (Figure 5). Thus after a 6 hour incubation with 1 mM glutamate, degenerated and dead cells were clearly visible in the culture (Figure 5B), the cellular extensions (dendrites) seen in untreated cortical cells (Figure 5A) were retracted, and cells appeared rounded. In the presence of calpain inhibitors (12.5 μM ALLN) or caspase-3 specific inhibitor (50 μM z-DEVD), dendrites were retained and cells had the appearance of untreated normal cells (Figures 5C and 5D), however, some degenerated and dead cells were also visible. Activation of caspase-3 was also detected with a highly specific active caspase-3 antibody raised against amino acids 163 to 175 (p18 subunit) of murine caspase-3 (data not shown). No change in caspase-3 activity was found in HT22 exposed to various concentrations of glutamate for up to 8 hours, while activation of caspase-3 significantly increased in glutamate-treated cells in primary cortical cells as observed previously by us [6].

### Effects of calpain and caspase inhibitors on glutamate-induced DNA fragmentation

**Figure 6:**
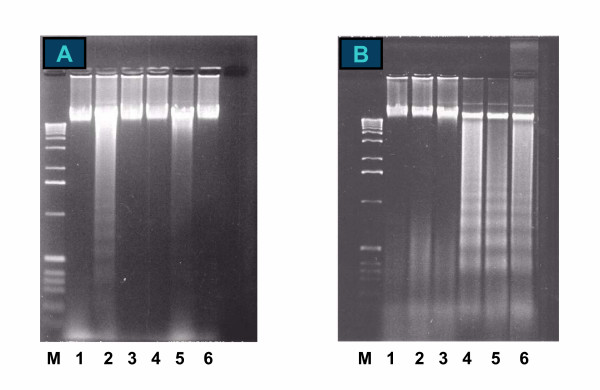
**Effect of calpain and caspase inhibitors on glutamate-induced DNA fragmentation (DNA ladder) in HT22 cells**. HT22 cells were treated with 8 mM glutamate in the presence or absence of calpain inhibitors 25 μM ALLN and 100 μM PD150606, and caspase inhibitors 100 μM z-DEVD and 100 μM z-VAD. A: HT22 cells were treated with glutamate in the presence of calpain inhibitors for 8 hrs. Lane 1 = untreated control cells; Lane 2 = glutamate alone; Lane 3 = glutamate + PD150606; Lane 4 = PD150606 alone; Lane 5 = glutamate + ALLN; Lane 6 = ALLN alone. B: HT22 cells were treated with glutamate in the presence of caspase inhibitors for 16 hrs. Lane 1 = untreated control cells; Lane 2 = z-DEVD alone; Lane 3 = z-VAD alone; Lane 4 = glutamate alone; Lane 5 = glutamate + z-DEVD; Lane 6 = glutamate + z-VAD; M: 100 bp DNA marker. Gel electrophoresis was carried out as described under "Methods" and the gels were stained with ethidium bromide.

To further investigate the effects of calpain and caspase-3 inhibitors on glutamate-induced programmed cell death (apoptosis), their effects on glutamate-induced characteristic DNA fragmentation were determined. HT22 cells were harvested after 8 hours or 16 hours of glutamate (8 mM) exposure in the presence or absence of calpain inhibitors or caspase inhibitors. Total DNA was extracted, purified and subjected to agarose gel electrophoresis. The results indicate that untreated cells did not display any DNA fragmentation (Figures 6A and 6B, Lane 1). In contrast, (8 mM) glutamate treatment resulted in characteristic DNA fragmentation or laddering (Figures 6A, Lane 2) that was associated with an increase in cell death. Addition of optimal doses of calpain inhibitors, 100 μM PD150606 or 25 μM ALLN, reduced or blocked glutamate-induced DNA fragmentation, (Figure 6A, Lanes 3 and 5). In contrast, in the presence of caspase inhibitors, (8 mM) glutamate-induced DNA fragmentation (Figure 6B, Lane 4) was not prevented by either the caspase-3 specific inhibitor, 100 μM z-DEVD (Figure 6B, Lane 5) or 100 μM z-VAD (Figure 6B, Lane 6) for up to 16 hours. In the absence of glutamate, none of the inhibitors alone (Figures 6A and 6B) had any effect on DNA fragmentation.

### Determination of caspase and calpain activation

Activation of calpain and caspase-3 after glutamate exposure in the primary cortical cells and HT22 cells was assessed by Western blot analysis by examining proteolysis of α-fodrin (α-II-spectrin) utilizing an epitope-specific antibody directed against calpain or caspase-3 cleavage site of α-fodrin. Cleavage of α-fodrin leading to formation of 150/145 kDa breakdown fragments is a well-recognized marker for the calpain-generated protein breakdown product (BDP) and formation of 120 kDa fragment for the caspase-3 specific (BDP). Antibodies raised against this epitope of α-fodrin have been shown previously to be specific for calpain-cleaved α-fodrin breakdown products 145 kDa or/and 150 KDa (145/150 kDa BDPs) and caspase-produced α-fodrin breakdown products 120 KDa (120 KDa BDP) [21,42]. The presence of α-fodrin BDPs 145/150 and BDP 120 would suggest that calpain and caspase-3 are activated under apoptotic conditions [43].

**Figure 7:**
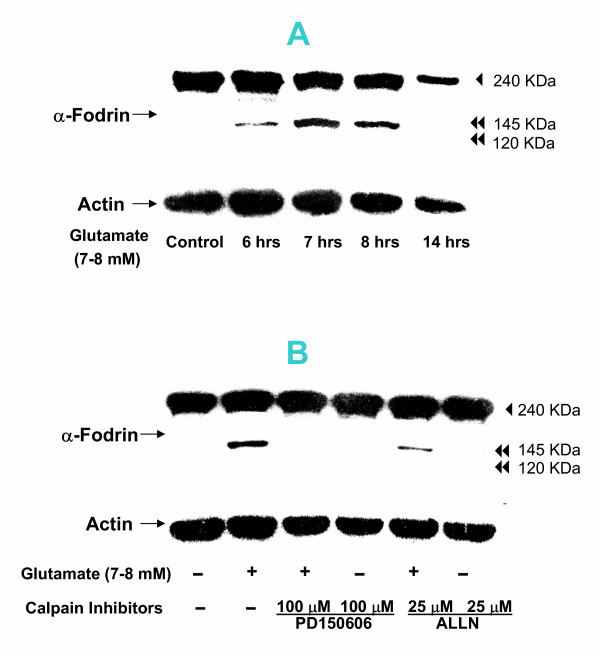
**Glutamate-induced the cleavage of α-fodrin in HT22 cells and inhibition by calpain inhibitors as detected by Western blot analysis**. A: HT22 cells were treated with 7–8 mM glutamate for the indicated times. Control sample showed intact α-fodrin protein (240 kDa). Accumulation of calpain-mediated 145 kDa BDP was detected after 6 hrs of glutamate treatment, but was not detectable after 24 hrs of treatment. There is no evidence showing caspase-3-produced 120 kDa BDP. B: HT22 cells were treated with 7–8 mM glutamate in the presence of calpain inhibitors, 100 μM PD150606 and 25 μM ALLN, for 8 hrs. Both calpain inhibitors could block or reduce calpain-mediated 145 kDa BDP. Treatment with calpain inhibitors alone had no effect on proteolysis of α-fodrin. Each experiment was repeated 3 times and a representative example of the Western blot is shown.

In HT22 cells, untreated cells contained the 240 KDa intact α-fodrin band and following treatment with glutamate, the 240 KDa band was cleaved into 145 KDa BDP, but not the α-fodrin 120 KDa BDP in this cell line. As shown in Figure 7A, glutamate induced calpain activity in a time-dependent manner. Thus, calpain activity began to increase at 6 hours after 7–8 mM glutamate exposure, which preceded glutamate-induced LDH release and apoptotic cell death, and was robustly activated by 7–8 hours. After a 14–16 hour treatment, degradation of native α-fodrin was observed and BDPs were less noticeable. The addition of calpain inhibitors (100 μM PD150606 or 25 μM ALLN) completely blocked or reduced glutamate-induced formation of 145 kDa BDPs (Figure 7B), which is consistent with the observation that they protect against glutamate-induced DNA fragmentation. These data further demonstrate that calpain protease is indeed activated and mediates glutamate-induced apoptotic cell death via a caspase-independent pathway in HT22 cells.

**Figure 8:**
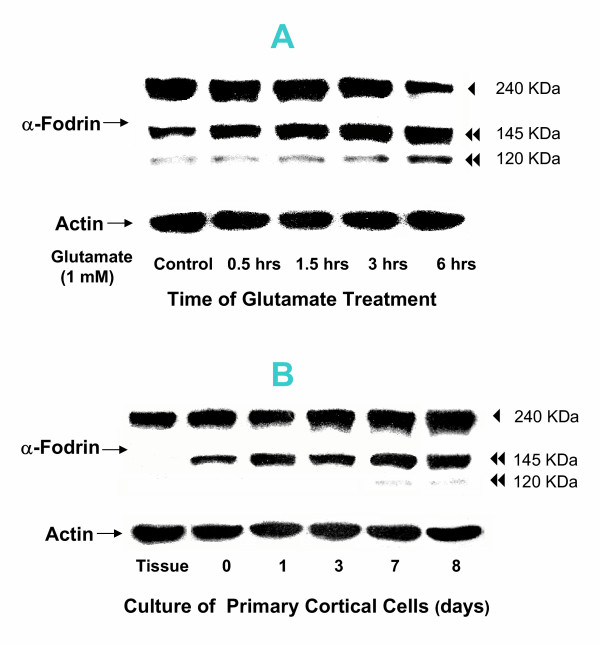
**Glutamate-induced the cleavage of α-fodrin in primary cortical cells**. A: primary cortical cells isolated from 18 day old fetal rat brains were cultured for 7–8 days and then cells were treated with 1 mM glutamate for the indicated times. Western blot analysis showed that calpain-mediated 145 kDa BDP and caspase-3-mediated 120 kDa BDP could be detected in untreated control cells. Accumulation of 145 kDa BDP was evident at 0.5 hr earlier than that of 120 kDa BDP, with greater accumulation of both breakdown products occurring at 6 hrs. B: primary cortical cells were cultured for the indicated days and the cleavage of α-fodrin was detected by Western blot. Tissue sample only contained the intact 240 kDa α-fodrin protein but no cleavage products. Cleavage of the 240 kDa α-fodrin protein into 145 kDa BDP began at day 0 of culture (dissociated cells prior to seeding on plates) and the 120 kDa BDP of α-fodrin was only found after 7 days of culture. Each experiment was repeated 3 times and a representative example of the Western blot is shown.

**Figure 9:**
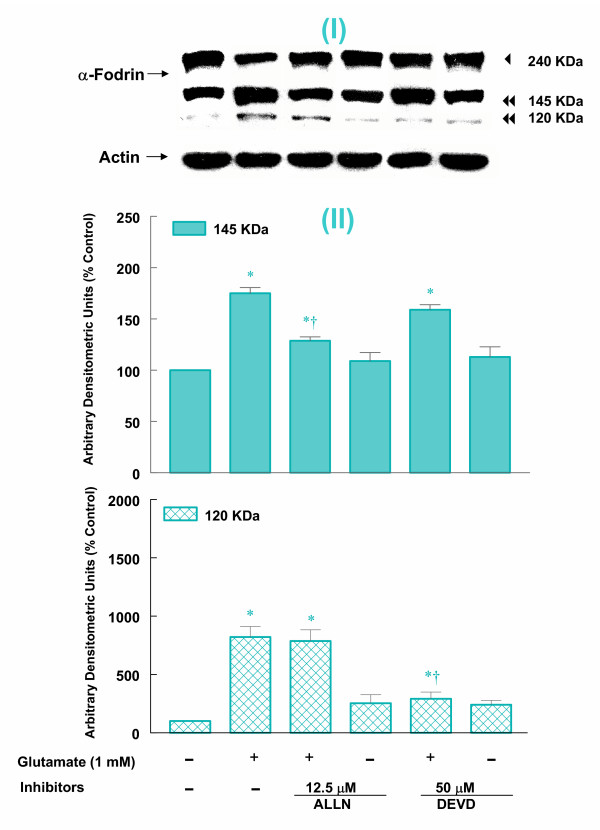
**Effect of calpain and caspase-3 inhibitors on glutamate-induced cleavage of α-fodrin in primary cortical cells**. Primary cortical cells isolated from 18 day old fetal rat brains were cultured for 7–8 days, and then cells were treated with 1 mM glutamate alone or in the presence of calpain inhibitor 12.5 μM ALLN and caspase-3 inhibitor 50 μM z-DEVD for 6 hrs. (I): representative Western blots showing calpain and caspase-3- mediated proteolysis of α-fodrin and inhibition by protease inhibitors. (II): semi-quantitative analysis of calpain and caspase-3-mediated proteolysis of α-fodrin. Accumulation of breakdown products was assessed by Western blot analysis as described under "Methods". Actin was used as a loading control. The bars depict densitometric analysis of Western blots from at least three experiments (± SEM) *P < 0.05 compared to control; **†***P *< 0.05 compared to glutamate alone.

In contrast to HT22 cells, cleavage of α-fodrin (240 kDa) protein into 145 kDa and 120 kDa BDPs was found in untreated cells (7–8 days of culture) (Figure 8A and 8B). Similarly, glutamate treatment resulted in an increase in the activity of calpain and caspase-3 in a time-dependent manner. Formation of 145 kDa BDPs by calpain began to increase earlier (0.5 hour) than the caspase-mediated 120 kDa BDP (3 hours), with greater accumulation of both breakdown products (145 kDa and 120 kDa) after 6 hours (Figure 8A). To further investigate the α-fodrin breakdown patterns produced by calpain or caspase-3, the effects of calpain and caspase inhibitors on glutamate-induced cleavage of α-fodrin were investigated by immunoblotting. The presence of calpain inhibitor 25 μM ALLN reduced the formation of BDPs 145 kDa in primary cortical cells exposed to glutamate, but it had no effects on 120 kDa BDP120 (Figure 9). However, the addition of caspase-3 specific inhibitor 50 μM z-DEVD completely inhibited the formation of 120 kDa BDP, but had no effect on the formation of 145 kDa BDPs (Figure 9).

We have previously reported that primary cortical cells in culture for longer than 2 days display characteristic DNA fragmentation. To further detect whether cellular mechanisms mediating this apoptotic process involve activation of these proteases, cleavage of α-fodrin was measured by using Western blot analysis with cells from fresh tissue, day 0, day 1, day 3, days 7 and 8 in culture (Figure 8B). Calpain-mediated cleavage of α-fodrin protein into 145 KDa BDPs could be seen on day 0 of culture (dissociated cells prior to culture) prior to the initiation of DNA fragmentation (2 days). The activation further increased up to 7 days. Caspase-3-dependent 120 KDa BDP was detectable only after 7 days of culture. These data indicate that apoptosis occurs during the development of the neuronal cells (increasing days in culture) and is also associated with calpain and caspase-3 activation. The results further indicate that activation of calpain precedes the presence of apoptosis characterized by DNA laddering and caspase-3 activity and suggest that calpain protease may be the first protease in mediating neuronal apoptosis.

### Effect of estrogens on the activation of calpain and caspase-3

**Figure 10:**
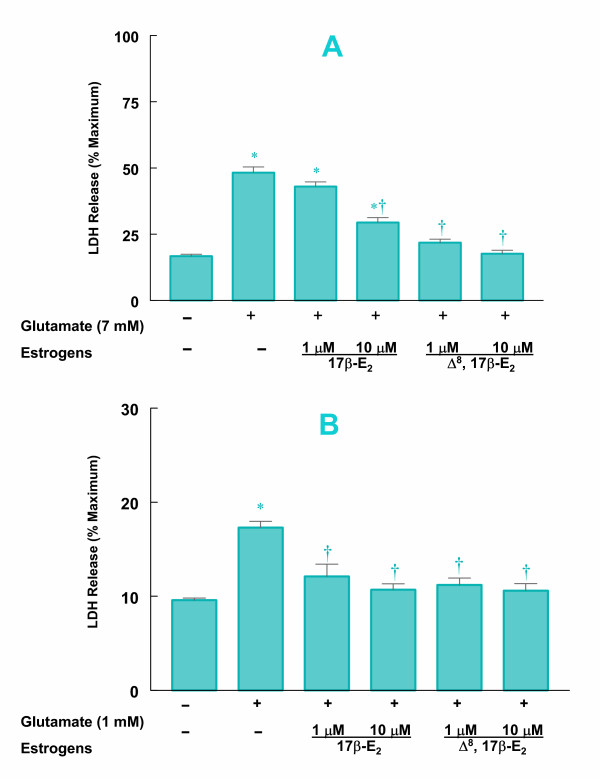
**Effects of estrogens, 17β-estradiol and Δ^8^,17β-estradiol, on glutamate-induced cell death in HT22 cells and primary cortical cells**. A: HT22 cells were treated with 7 mM glutamate in the presence or absence of 1 μM and 10 μM 17β-estradiol or Δ^8^,17β-estradiol for 8 hrs. Cell death was measured by LDH release assay. B: primary cortical cells isolated from 18 day old fetal rat brains were cultured for 7–8 days and were treated with 1 mM glutamate in the presence or absence of 1 μM and 10 μM 17β-estradiol or Δ^8^,17β-estradiol. LDH release was measured after 6 hrs of treatment. Data are the mean ± SEM values from at least three experiments *P < 0.05 compared to control; **†***P *< 0.05 compared to glutamate alone.

**Figure 11:**
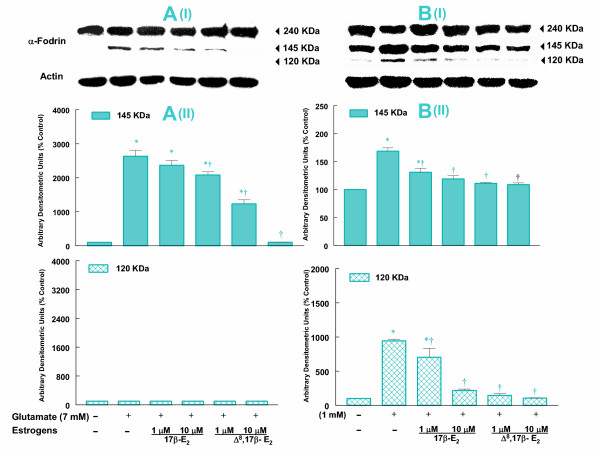
**Effect of estrogens, 17β-estradiol and Δ ^8^,17β-estradiol, on glutamate-induced cleavage of α-fodrin in HT22 cells and primary cortical cells**. A: HT22 cells were treated with 7 mM glutamate in the presence or absence of 1 μM and 10 μM 17β-estradiol or Δ^8^,17β-estradiol for 8 hrs. B: primary cortical cells isolated from 18 day old fetal rat brains were cultured for 7–8 days and treated with 1 mM glutamate in the presence or absence of 1 μM and 10 μM 17β-estradiol or Δ^8^,17β-estradiol for 6 hrs. A, B: (I): representative Western blots showing calpain and caspase-3-mediated proteolysis of α-fodrin and inhibition by estrogens. A, B: (II): semi-quantitative analysis of calpain and caspase-3-mediated proteolysis of α-fodrin. Accumulation of breakdown products was assessed by Western blot analysis as described under Figure 9. Actin was used as a loading control. The bar depict densitometric analysis of Western blots from at least three experiments (± SEM) *P < 0.05 compared to control; **†***P *< 0.05 compared to glutamate alone.

We have previously reported that estrogens, 17β-estradiol (17β-E_2_) and Δ^8^,17β-estradiol (Δ^8^,17β-E_2_), protect against glutamate-induced cell death in a dose-dependent manner. HT22 cells were treated with 7 mM glutamate for 7–8 hours in the presence or absence of 1 μM and 10 μM 17β-E_2 _or Δ^8^,17β-E_2_. Cell death was measured by LDH release assay and the results are summarized in Figure 10A. Both 17β-E_2 _and Δ^8^,17β-E_2 _inhibited glutamate-induced cell death, with Δ^8^,17β-E_2 _being more potent. Thus, in the presence of 17β-E_2_, only 10 μM 17β-E_2 _could significantly reduce cell death compared to glutamate alone (Figure 10A). However, the presence of Δ^8^,17β-E_2_, 1 μM or 10 μM, completely inhibited cell death and returned the release of LDH to control levels. In contrast, in primary cortical cells exposed to 1 mM glutamate for 6 hrs, 1 μM or 10 μM 17β-E_2 _or Δ^8^,17β-E_2 _could completely protect against cell death (Figure 10B). Following measurement of LDH, inhibition by estrogens of glutamate-induced cleavage of α-fodrin in both cell types was determined by Western blot analysis. In HT22 cells, the presence of 10 μM 17β-E_2 _and 1 μM Δ^8^,17β-E_2 _in 7 mM glutamate for 8 hours significantly decreased glutamate-induced cleavage of α-fodrin into 145 KDa BDP by 21% and 51%, respectively, compared to glutamate alone (Figure 11A). However, 10 μM Δ^8^,17β-E_2 _totally inhibited the formation of 145 KDa BDP. Moreover, in primary cortical cells exposed to glutamate (1 mM) in the presence of estrogens (1 μM and 10 μM 17β-E_2 _or Δ^8^,17β-E_2_) for 6 hours, estrogens not only inhibited calpain-mediated formation of 145 KDa BDP, but also reduced caspase-3-mediated formation of 120 KDa BDP (Figure 11B). Thus, 1 μM 17β-E_2 _significantly reduced breakdown products of 145 and 120 KDa compared to glutamate alone. Similarly, 10 μM 17β-E_2 _and 1 μM Δ^8^,17β-E_2 _could completely prevent activation of calpain and caspase-3 proteases and totally block cleavage of α-fodrin. These data indicate that estrogens protect against glutamate-induced apoptotic cell death by inhibiting both calpain and caspase-3 activity in neuronal cells, with Δ^8^,17β-E_2 _being more potent.

### Effects of glutamate and estrogens on protein levels of apoptosis inducing factor (AIF) in different cell fractions from two cell types

**Figure 12:**
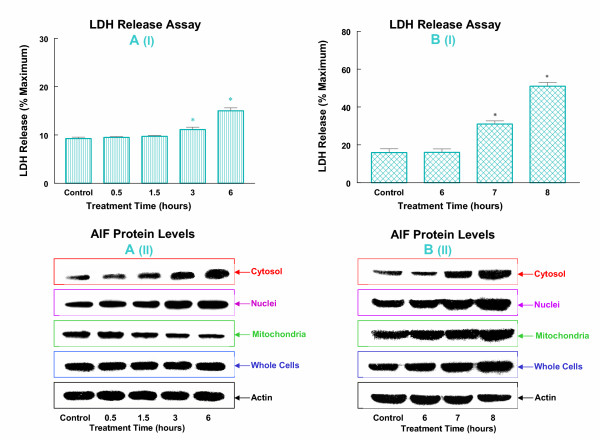
**Effect of glutamate on LDH release and AIF protein levels in subcellular fractions in HT22 cells and primary cortical cells**. A: primary cortical cells were isolated as described under Figure 11 and were treated with 1 mM glutamate at the indicated times. B: HT22 cells were treated with 7–8 mM glutamate at the indicated times. A, B: (I): cell death were measured by LDH release assay. Data are the mean ± SEM values from at least three experiments *P < 0.05 compared to control; A, B: (II): subcellular proteins were isolated from cytosol, nuclei, mitochondria and whole cells as described under "Methods", and then AIF protein levels were detected by Western blot analysis. Actin was used as a loading control.

**Figure 13:**
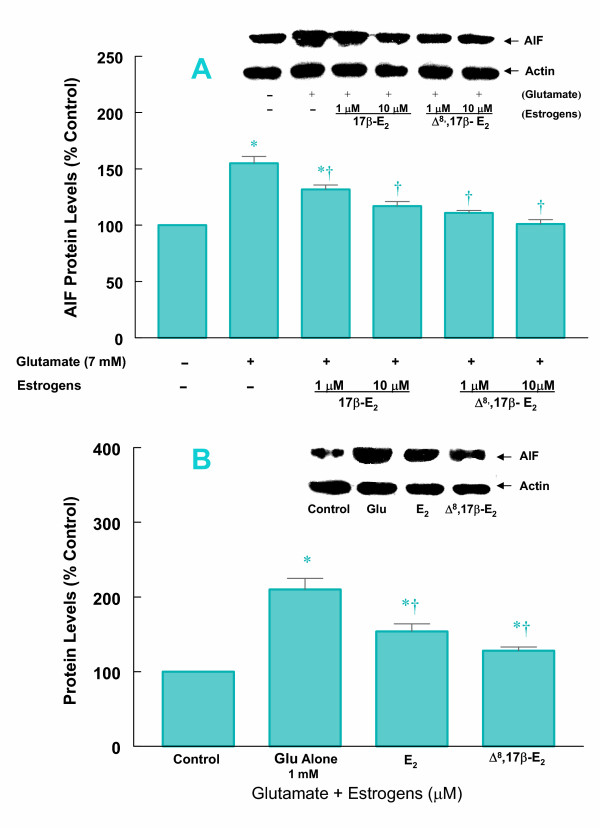
**Effect of estrogens, 17β-estradiol and Δ^8^,17β-estradiol, on AIF protein levels during glutamate-induced cell death in HT22 cells and primary cortical cells**. A: HT22 cells were cultured and were treated as described under Figure 11. Cells were then harvested and total protein was extracted. AIF protein levels were determined by Western blot analysis. The bars depict densitometric analyses from at least three experiments (± SEM). *P < 0.05 compared to control; **†***P *< 0.05 compared to glutamate alone. B: cortical cells were isolated as described under Figure 11 and were treated with 1 mM glutamate in the presence or absence of 1 μM 17β-estradiol or Δ^8^,17β-estradiol for 6 hrs. Cytosol protein was isolated and AIF protein levels were detected as described under "Methods". Actin was used as a loading control.

Following the determination of calpain and caspase-3 protease's involvement in apoptotic cell death, regulation of mitochondrial apoptotic effector AIF in cells undergoing apoptosis was determined in primary cortical cells and HT22 cells. Protein levels in cytosol, mitochondria, nuclei and whole cell lysates were detected by Western blot analysis. In cortical cells, treatment with glutamate (1 mM) resulted in an increase in AIF protein levels in the cytosol and nuclei in a time dependent-manner (Figure 12A). Concomitant with these changes, the levels of AIF protein decreased in the mitochondria and no changes were found in whole cell lysates. The elevation of cytosolic and nuclear AIF and its reduction in the mitochondria were associated with an increase in cell death. These results indicate that AIF protein is released or translocates from mitochondria into cytosol and nuclei in primary cortical cells undergoing apoptotic cell death following exposure to glutamate. However, exposure of HT22 cells to glutamate (7–8 mM) enhanced AIF protein levels in all subcellular fractions of cells in a time-dependent manner that is also associated with glutamate-induced cell death. As shown in Figure 12B, AIF protein levels were increased not only in the cytosol and nuclei but also in the mitochondria and whole cell lysates. These data demonstrate that glutamate can upregulate AIF protein levels in HT22 cells. Whether translocation of AIF is involved in this process requires further study. The presence of estrogens, 1 μM and 10 μM 17β-E_2_or Δ^8^,17β-E_2_, decreased glutamate-induced upregulation of AIF in HT22 cells and reduced glutamate-induced AIF release from mitochondria into cytosol in primary cortical cells. In HT22 cells, 1 μM 17β-E_2 _significantly decreased AIF protein levels in whole cell lysates by 40% compared to glutamate alone. Furthermore, 10 μM 17β-E_2 _and 1 μM Δ^8^,17β-E_2 _could reverse glutamate-induced levels of AIF to control values (Figure 13A). Similarly in primary cortical cells, 1 μM 17β-E_2 _and Δ^8^,17β-E_2 _could significantly reduce the release of mitochondrial AIF in cytosol by 30% and 50%, respectively compared to glutamate alone (Figure 13B).

## Discussion

The objective of this study was to investigate the cellular mechanisms involved in glutamate-induced apoptotic cell death in neuronal cells. We used the mouse hippocampal cell line HT22 and primary fetal rat cortical cells to demonstrate that glutamate can induce neuronal apoptotic cell death by apoptotic mechanisms and that the process can be reversed or inhibited by equine estrogens such as 17β-E_2 _or Δ^8^,17β-E_2_. The results further indicate that glutamate-induced apoptotic cell death involves the activation of apoptotic proteases calpain and caspase-3 as well as upregulation and/or translocation of apoptosis-inducing factor (AIF) from mitochondria into cytosol and nuclei, where it causes nuclear condensation and apoptosis by a caspase-independent pathway [44,45]. Our results reveal that in these two cell types, primary cortical cells and the HT22 cell line, cells display different patterns in regulating specific proteins involved in apoptosis. Thus, in primary cortical cells, glutamate-induced apoptotic cell death is associated with activation of calpain and caspase-3, which is in agreement with studies using different stimuli such as staurosporine [46], and release of apoptosis-inducing factor (AIF) from mitochondria into cytosol and nuclei. However in the mouse hippocampal cell line HT22, glutamate-induced apoptotic cell death involved calpain protease activity and upregulation of AIF protein, but not caspase-3 activity. Taken together, our data demonstrate that glutamate-induced apoptotic cell death in neuronal cells could be mediated by the apoptotic proteases calpain and caspase-3 as well as mitochondrial apoptosis effector AIF via a caspase-dependent or caspase-independent apoptotic pathway that depends on the cell type. In HT22 cells, caspase-3 protein was not detectable and glutamate-induced cell death occurred without elevation of caspase-3 activity (data not shown). Caspase inhibitors, either the specific caspase-3 inhibitor DEVD or the pancaspase inhibitor z-VAD, are not capable of protecting against glutamate-induced cell death in HT22 cells, while they do inhibit glutamate-induced cell death in primary cortical cells. In addition, glutamate-induced DNA fragmentation (ladder) can be reduced or blocked by calpain inhibitors but not by caspase inhibitors in this cell line. These results strongly indicate that glutamate-induced apoptotic cell death in HT22 cells is executed through a caspase-independent pathway as has been observed in platelets [7].

Caspase-3 is considered a key executioner in the apoptotic cell death cascade and shares numerous substrates with Ca^2+^-dependent protease calpain such as cytoskeletal protein α-fodrin in which they cleave different epitope sites once activated [24]. Though they both are capable of executing apoptotic cell death, they appear to play different roles in this process. Our results show that immunoblots probed with an anti-α-fodrin monoclonal antibody could detect intact α-fodrin (MWr = 240 KDa) and 145 KDa and 120 KDa BDPs [47]. Treatment with glutamate induced accumulation of both 145 KDa and 120 KDa BDPs in primary cortical cells, which could be reduced with both calpain inhibitor (ALLN) and caspase-3 inhibitor (z-DEVD-fmk). Thus, calpain inhibition specifically protects against glutamate-induced production of 145 BDP and caspase-3 inhibition inhibits production of 120 BDP. There were no synergetic effects on glutamate-induced apoptotic cell death when these protease inhibitors were combined. These studies indicate that both calpain and caspase-3 are indeed activated, and their activation occurs sequentially and independently after cells undergo apoptosis, suggesting they may execute cell death to some extent by distinct mechanisms in primary cortical cells. There is no evidence showing that they have overlapping roles in cell death as described previously in neuronal or non-neuronal cells [43]. Moreover, in HT22 cells, only calpain-mediated α-fodrin 145 KDa BDP appeared after glutamate treatment, but 120 KDa BDP generated specifically by caspase-3 [47,48] could not be detected. Furthermore, glutamate-induced cleavage of α-fodrin to 145 BDPs could be reduced or blocked by calpain inhibitors, further indicating that glutamate-induced apoptosis is mediated by a calpain cascade via a caspase-independent pathway in HT22 cells. Additional evidence for calpain's involvement in apoptotic cell death in central nervous system is supported by recent studies examining *invivo*traumatic brain injury TBI [49] and ischemia in the rat heart [50], in which calpain-mediated cleavage of α-fodrin and DNA fragmentation have been detected following these insults.

Calpain is a calcium-dependent neutral protease with two isoenzyme forms, μ-calpain and m-calpain, distinguished by their *invitro*calcium requirements. Calpain exists as a proenzyme in an inactive form in cytosol where the normal range of Ca^2+ ^concentration is 50–100 nM in resting cells [25,51]. An elevation of cytoplasmic free Ca^2+ ^concentration triggers calpain activation, which also depends upon the amount of calcium and site of activation. μ-Calpain is activated in the presence of low micromolar levels of Ca^2+^, and m-calpain requires millimolar Ca^2+ ^levels for its activation. After calpain is activated, it cleaves many cytoskeletal proteins and signaling molecules such as fodrin, filamine, neurofilament protein tau and tubulin [24,51]. Fodrin (α II spectrin) was found to be a specific calpain substrate [52,53]. In our studies, a calpain-mediated cleavage fragment of α-fodrin was observed in two cell types exposed to glutamate, as seen in the dystrophic neuritis and senile plaques in Alzheimer's diseased brains [54], suggesting that calpain could be a major apoptotic protease involved in the pathogenesis of neurodegenerative diseases.

It has been reported that calpain could be activated in both necrotic and apoptotic conditions as observed in various neurological and neurodegenerative disorders, such as cerebral ischemia and glutamate toxicity [55,56], while caspase-3 is only activated in neuronal apoptosis [44,57-59]. Nevertheless, in our model systems, glutamate-induced cell death in HT22 cells was found to involve the mechanism of apoptosis (programmed cell death) characterized by the presence of DNA fragmentation (DNA ladder) and morphological cell changes, and this process could be attenuated by calpain inhibitors and not caspase inhibitors. Thus, in cortical cells, both 145 KDa and 120 KDa BDPs were formed in untreated cells and cells treated with glutamate, which was also associated with DNA fragmentation (data not shown), and calpain-mediated 145 KDa BDP accumulated to a greater extent than 120 BDP. Furthermore, activation of calpain precedes that of caspase-3, glutamate-induced LDH release (cell death) and DNA fragmentation in these cells. Taken together, our results strongly suggest that calpain, rather than caspase-3, plays a critical role in mediating apoptosis characterized by oligonucleosomal DNA fragmentation (DNA ladder) and is a key apoptotic executioner in neuronal apoptosis, especially in HT22 cells. Whether calpain activity is associated with necrosis needs to be investigated.

We have previously reported that glutamate induces cytochrome c release from the mitochondria into the cytoplasm where it activates caspase-3 and causes apoptotic cell death in primary cortical cells via a caspase-dependent pathway [6]. In the present paper, we show that apoptosis inducing factor (AIF), normally confined in the mitochondria, is also released into the cytosol and nuclei in primary cortical cells undergoing apoptosis and upregulation of AIF also occurs in HT22 cells after glutamate treatment. In contrast to cytochrome c, AIF acts in a caspase-independent fashion [19,60]. AIF is a ubiquitously expressed flavoprotein with significant homology to bacterial oxidoreductases and has NADH oxidase activity [17]. Under normal circumstances, transcription and translation of nuclear AIF gene give rise to a precursor molecule (67 KDa) that carries a putative mitochondrial localization sequence in its NH_2 _terminus [60]. Upon import into the mitochondrial intermembrane space, this 100 amino acid precursor is cleaved by a local peptidase leading to generation of the mature AIF molecule (57 KDa). When apoptotic cell death is induced, AIF translocates through the outer mitochondrial membrane to the cytosol and nucleus, where it leads to nuclear chromatin condensation and a large-scale DNA fragmentation (high molecular weight DNA fragments) and apoptosis in a caspase-independent manner [10,11]. Recent studies show that microinjection of mature AIF and precursor AIF can both induce chromatin condensation and cell death [61,62]. It was further found that transfection enforced overexpression of the wild type precursor AIF (precursor AIF protein) also diminishes the permeability of the mitochondrial membrane (ΔΨ_μ_), induces AIF release from the mitochondria and causes apoptosis. Based on these studies, glutamate-induced overexpression of AIF in HT22 cells might also trigger AIF release or translocation from the mitochondria into the cytosol and nuclei. This process needs to be further elucidated. Taken together with previous studies, our data indicate that glutamate exerts its toxic action through two parallel pathways: a caspase-dependent pathway mediated by caspase-3, the final apoptotic effector, and a caspase-3 independent apoptotic pathway involving the upregulation of AIF and/or mitochondrial AIF release as well as calpain-modulated effects (cell death).

We have previously reported that a number of equine estrogens, which are components of the drug CEE used extensively for management of vasomotor symptoms and osteoporosis in postmenopausal women, are potent antioxidants and protect neuronal cells against cell death induced by oxidized LDL or glutamate [32,33,35,63-65]. Our current findings indicate that estrogens prevent glutamate-induced cell death by reducing the upregulation and/or translocation of AIF from the mitochondria into the cytosol as well as by attenuating activity of calpain and caspase-3 in primary cortical cells and HT22 cells, with Δ^8^, 17β-estradiol being more potent than 17β-estradiol. Although neurotoxic effects of oxidized LDL and glutamate can be inhibited differentially by various estrogens, [32,33,35], the data presented in this paper, to our knowledge, represents the first comprehensive analysis of the involvement of multiple apoptotic proteins in glutamate-induced apoptosis in different neuronal cells that can be differentially inhibited by equine estrogens. Recent studies have shown that estrogen can decrease calpain activity in rat C6 glial cells treated with H_2_O_2 _and in ischemic muscle [66,67]. The regulation by estrogens on the translocation of apoptotic effectors from the mitochondria into the cytosol such as cytochrome c and the activation of caspase-3 has been discussed in previous reports in which estrogens might directly modulate the mitochondrial Ca^2+ ^content and mitochondrial trans-membrane potential [6]. As the estrogen receptor has been shown to be a substrate of calpain, estrogen-mediated reduction of calpain activity may prevent degradation of the ERs, and enable receptor-mediated events to proceed by genomic mechanism [68]. Whereas in HT22 cells lacking estrogen receptors, estrogen effects are most-likely mediated to some extent by non-genomic mechanism [4,69]. It may also be related to an effect on voltage-gated Ca^2+ ^channels and reduced post-injury influx of Ca^2+ ^[70]. It has been reported that estrogen treatment can attenuate Ca^2+ ^influx in cultured neurons [71]. However, our earlier findings indicate that estrogens protected primary cortical cells against glutamate induced excitotoxicity by a mechanism that appears to be independent of Ca2+ influx [13]. In this study in primary cultures of cortical neurons, we investigated the glutamate-induced excitotoxicity by exposing the neurons to μM concentration of glutamate for only 20 minutes. The results from this study indicated that glutamate-induced cell death occurs through NMDA receptors and NOS-linked mechanism independent of Ca2+ influx and this form of cell death was also prevented by estrogens. A recent study [72] has confirmed our latter observations in neuronal cells derived from fetal rat brains, however, these investigators also used middle aged and old rats neurons where there was a profound loss of calcium homeostasis more so in the older age group. These observations indicate that neurons from older rats are more sensitive to glutamate toxicity, however, as in neurons from fetal brains, estrogens were also able to protect these older neurons. Whether the role of calpain, caspase-3 and AIF is altered in cortical neurons derived from older animals remains to be investigated.

Pre-treatment of these neurons with 17β-estradiol prior to exposure to glutamate attenuate and delay the adverse effects on intracellular Ca^2+ ^homeostasis. Whether the role of calpain, caspase-3 and AIF is altered in cortical neurons derived from older rat brains remains to be investigated. Our observations further suggest that the neuroprotective effects of estrogens can involve both genomic and non-genomic mechanisms that depend on the neuronal cell type and the cytotoxin used to induce apoptosis.

Recently, Bano et al [73] reported that the Na^+^/Ca^2+ ^exchanger (NCX) which under normal physiological conditions, transports Na^+ ^ions into the cell and Ca^2+ ^out, is destroyed by Ca^2+^-activated calpain under ischaemic conditions. This proteolytic inactivation of NCX could allow further accumulation of Ca^2+ ^or delay its elimination, that ultimately results in the destruction of the neurons. Whether glutamate induced Ca^2+ ^influx and the activation of calpain observed in our neuronal cells also results in the inactivation of NCX that can be prevented by estrogen remains to be investigated.

Although our present data and those of a number of previous studies [20,21,23,24,26-29] indicate that calpains play a critical role in mediating apoptotic cell death, others indicate that in cerebellar granule cells and rat fetal hippocampal neurons activation of NMDA receptors is necessary for calpain-1 activation but no association between calpain activation and glutamate-induced cell death was observed [74-76]. Differences between these observations could be due to several factors such as cell type, primary cell cultures or stable cell line cultures, tissue culture conditions, source of cells (fetal, middle age or adult brains), age of cultures, purity of cultures concentration and duration (minutes to hours) of glutamate exposure, nature of the neurotoxin used, and extent of Ca^2+ ^influx [13,74-76]. Along with these, a number of additional differences have been indicated [76]. Further detailed studies are needed to delineate the role of calpains in glutamate-induced apoptotic neuronal cell death in clearly defined model systems. It is also important to recognize that at least two distinct pathways for glutamate-induced cell death have been described: the excitotoxic pathway and the oxidative pathway. The excitotoxic pathway involves the overactivation of glutamate receptors that leads to both rapid and slowly triggered cytotoxic events. The rapid effects involve the activation of the NMDA receptor and leads to a large Ca2+ influx that is detrimental to cell viability. The oxidative pathway involves the breakdown of the glutamate-cystine antiporter and a drop in glutathione levels that allows for aberrant formation of reactive oxygen species (ROS) that are neurotoxic [77-79]. In most of our studies, we and a number of other investigators have used higher concentration (mM) and exposed the cultures to longer exposure times (hours vs minutes) to glutamate. Under these conditions, we usually get 30 to 50% cell death. This cell death occurs most likely by mechanism distinct from excitotoxicity induced by acute exposure of neurons to μM concentrations of glutamate. We think as indicated in the first sentence in the background it is the high concentrations (mM) of the excitatory neurotransmitter glutamate that accumulates in the aging brain that may play a role in neurodegeneration associated with normal aging. In contrast, in acute trauma to the brain, excitotoxicity may be the key factor. In our recent study [13] in primary cultures of cortical neurons, we investigated the glutamate-induced excitotoxicity by exposing the neurons to μM concentration of glutamate for only 20 minutes. The results from this study indicated that glutamate-induced cell death occurs through NMDA receptors and NOS-linked mechanism independent of Ca2+ influx and this form of cell death was also prevented by estrogens. Thus, depending on the objectives of the study, we have used different concentrations of glutamate. Important to note that in HT22 cells, NMDA receptors are absent and therefore this form of excitotoxicity may not occur in these types of neuronal cells.

Since in our study both NMDA receptor and estrogen receptor positive (primary cortical cells) and negative (HT22) cells were used, the results therefore indicate that at least two different mechanisms are involved in the activation of calpains. Which of these mechanisms is involved in neurodegenerative diseases such as Alzheimer's remains to be investigated. It is likely that in the aging brain, neurons are more sensitive to chronically high levels (mM) of glutamate that can result in the activation of calpains and the initiation of neurodegenerative process. Over time, the caspase-3 system is also activated leading to a higher degree of apoptotic neuronal cell death and progression of the disease process. The demonstration that equine estrogens can prevent the activation of both types of proteases, provides an avenue of developing new therapeutic interventions for the prevention of neurodegenerative diseases.

## Conclusion

Our present studies demonstrate that at least two mechanisms are involved in glutamate-induced apoptosis: a caspase-dependent pathway via a caspase-3 cascade and a caspase-independent pathway involving calpain protease and AIF-mediated apoptosis. Since HT22 cells lack caspase-3 protein and activity, glutamate-induced apoptosis is mediated via the caspase-independent pathway in this particular cell line. Our data also suggest that calpain, rather than caspase-3, plays a critical role in the neuronal intracellular pathway leading to glutamate-induced apoptosis. Our studies further indicate that glutamate-induced changes in these apoptotic proteins can be inhibited by estrogens, with Δ^8^,17β-estradiol, a novel equine estrogen being more potent than 17β-estradiol. To our knowledge, this is the first demonstration that glutamate-induced apoptosis involves regulation of multiple apoptotic effectors that can be inhibited by estrogens. These data provide an insight into the development of potential novel therapeutic approaches for the prevention of neurodegenerative disease with estrogens and calpain inhibitors.

## Methods

### Materials

All primary and secondary antibodies along with other reagents were purchased from commercial sources: primary antibodies, goat polyclonal antibody to AIF (*sc 9416*) and goat polyclonal antibody to Actin (*sc1616*) from Santa Cruz Biotechnology, Inc (Santa Cruz, Ca). Mouse monoclonal antibody to α-fodrin (FG 6090) (α II-spectrin, non-erythroid) from Affiniti Research Products (Hornby, ON). Estrogen receptor (ERα and ERβ) antibodies were generously donated by Drs Istvan Merchenthaler (Wyeth Inc) and Geoffrey Greene (University of Chicago). Secondary antibodies against mouse and goat were purchased from Sigma (Saint Louis, MI). SuperSignal^® ^West Pico Chemiluminescent Substrate was purchased from PIERCE (Rockford, IL). Caspase inhibitors, Z-Val-Ala-DL-Asp (OMe)-fluoromethylketone (Z-VAD-fmk) and Z-Asp (OMe)-Glu (OMe)-Val-DL-Asp (OMe)- fluoromethylketone (Z-DEVD-fmk) were purchased from Bachem Bioscience Inc (King of Prussia, PA), and calpain inhibitors, ALLN (Cat# 208719) and PD150606 (Cat# 513022) were purchased from Calbiochem (Missisauga, ON) the Cytotox 96 Non-radioactive Cytotoxicity Assay Kit (Promega G1780) was obtained from VWR (Toronto, ON). Neurobasal, B27, Hanks' buffered salt solution (HBSS) and antibiotics were from Gibco Life technologies (Burlington, ON). Other chemicals were of reagent grade and were obtained from VWR, Canada.

### Cell culture

The method used has been previously described in detail [6,37]. Briefly, eighteen-day-old pregnant Sprague-Dawley rats were sacrificed by cervical dislocation. Fetuses were delivered and fetal skulls were carefully opened and the meninges were removed. Two pieces of cortex (0.5–1.5 × 1–2 mm) were cut from the posterior dorsal surface from each of the fetuses and dispersed by mechanical dissociation in Hank's balanced salt solution (HBSS, Ca^2+ ^and Mg^2+ ^free). Cells thus obtained, were suspended in 2 × volume of HBSS with Ca^2+ ^and Mg^2+ ^and allowed to settle for 3 to 5 minutes. The supernatant was centrifuged at 1100 rpm (200 g) for 1 minute. The pellet was suspended in HBSS buffer and fresh cell suspensions were plated onto poly-L-lysine-coated 96-well plates (5 × 10^4^/well) or 6-well plates (1 × 10^6^/well) containing neurobasal medium supplemented with 2% B27, 0.5 mM L-glutamine, 25 μM glutamic acid, 120 mg/L penicillin and 260 mg/L streptomycin (NBC). The cells were then maintained at 37 C in a humidified incubator with 5% CO_2_/95% air in NBC. After day 1 of plating, the media was completely replaced and on subsequent days, 50% of the media was replaced. Experiments were performed on 8 day cultures in neurobasal medium containing 2% antioxidant free B27 supplement. Under these serum-free culture conditions, cells used are essentially pure (>95%) neuronal as demonstrated previously [37]. We confirmed the purity of our cultures by demonstrating the presence of neuron specific enolase (NSE) and the absence of glial fibrillary acid protein (GFAP). Almost all cells present in the primary cortical cultures stained positively for only NSE. These data clearly demonstrate the purity of our cultures. The details of the methods used have been described in our previous study [13]. The mouse hippocampal cell line (HT22) was grown as described previously [5]. HT22 cells were plated in 96-well plates (3 × 10^4^/well) or 6-well plates (3 × 10^5^/well) in Dulbecco's Modified Eagle Medium (DMEM) containing 4.5 g/L glucose, 5 g/L sodium bicarbonate, 10 mM Hepes, 120 mg/L penicillin, 75 mg/L streptomycin, 5% horse serum and 10% fetal bovine serum. The cells were incubated at 37 C in a humidified incubator with 5% CO_2_/95% air. After 24 hours, the medium was changed and cells were treated with glutamate in the presence or absence of various concentrations of caspase and calpain inhibitors or estrogens.

### Determination of glutamate-induced cell cytotoxicity

Cell death was estimated using a validated LDH (Lactate dehydrogenase) release assay as described previously [5,6]. The release of lactate dehydrogenase in the culture medium indicated loss of membrane integrity and cell death. LDH activity in the culture medium was determined using the non-radioactive cytotoxicity assay kit according to the manufacturer's protocol. The absorbance at 490 nm was measured in a microplate reader (Spectra MAX 340). In all experiments, cultures were also treated with 0.1% Triton X-100 to lyse; the cells and LDH levels measured under these conditions were taken as the maximal LDH release (100% cell death). The results are expressed as a percentage of maximum LDH release. We have previously established a strong correlation between the MTS (3 [4,5-dimethylthiazol-2yl]-5 [3-carboxymethoxyphenyl]-2H tetrazolium inner salt), cell proliferation (viability) assay and the LDH assay [80]. In the MTS assay, live cells once treated with the reagent are not further useable for other measurements. Since we wanted to use the same cells for Western blot analysis, the LDH assay was selected as in this assay, only the supernatant is used and the cells can be processed for other determinations.

### Western blot analysis

Protein levels of α-fodrin and apoptosis inducing factor (AIF) were determined by Western blot analysis using polyclonal and monoclonal antibodies raised against AIF and α-fodrin respectively, as described previously [5,6]. In brief, cells were washed twice with cold PBS, harvested using a cell scraper, and lysed in a cell lysis buffer (9 mM Na_2_PO_4_, 1.7 mM NaHPO_4 _and 150 mM NaCl, pH7.4), containing 1% Nonidet P-40 (Sigma, Toronto, Canada), 0.5% sodium deoxycholate, 0.1% SDS and 1 mM phenylmethylsulfonylfluoride for 20 minutes on ice. Cell lysates were centrifuged at 10,000 g at 4 C for 10 minutes. The protein concentration was determined by Bradford method (BioRad, Toronto, Canada). Cell lysates containing 10 to 20 μg protein were added to equal volume of 2 × reducing sample buffer (100 mM Tris-Cl pH 6.8, 200 mM dithiothretiol, 4% SDS, 20% glycerol, and 0.2% Bromophenol blue) and heated at 100 C for 3 minutes. The samples were separated by discontinuous 7% and 10% polyacrylamide gel electrophoresis under constant current (14–15 mA). Separated proteins were electrotransfered onto a Protan nitrocellulose membrane (Schleicher & Schuell Inc., Keene N.H). The blots were blocked with 5% non-fat milk in TBST (20 mM Tris-HCl pH 7.6, 137 mM NaCl, 0.05% Tween-20) at 4 C overnight and then incubated with primary antibody for 1–2 hours at room temperature, washed three times with TBST and incubated (1–2 hours) with appropriate horse radish peroxidase-conjugated secondary antibody. The membranes were washed three times with TBST and the immunoblots were visualized on X-ray films (Sigma) after exposure to enhanced chemiluminescence reagent (ECL), (Amersham, Toronto, Canada). Actin bands were monitored on the same blot to verify consistency of protein loading. Briefly, the immunoblots were stripped with TBST containing 0.04% sodium azide for 30 minutes at room temperature. The blots were probed with anti-actin primary antibody and secondary antibody (anti-goat) as described above. The molecular size of protein was determined by running pre-stained protein markers in an adjacent lane, omitting primary antibody as a procedural control. Each experiment was repeated at least three times. The band intensity was measured from scanned images using UN-SCAN IT Gel Automated Digitizing system, Version 5.1 (Silk Scientific, Inc. Orem, Utah, USA), software.

### RT-PCR assay

Total RNA were extracted from cortical cells by using RNeasy Mini Kit (Qiagen Inc, Mississauga, ON) according to the manufacturer's protocol. The concentration of total RNA was determined by reading the absorbance at 260 nm in a spectrophotometer. Semi-quantitative RT-PCR was performed to assess the expression of estrogen receptor mRNA (ERα and ERβ) with G3PDH as an internal control. Briefly, cDNA synthesis (RT) was performed with 1–1.5 μg total RNA in 30 μl RT mixture buffer containing 50 mM Tris-Cl, pH 8.5, 75 mM KCl, 3 mM MgCl, 10 mM DTT, 1 mM dNTP, 250 ng random primer, 3.5 units RNase inhibitor (Pharmacia), 200 units AMV reverse transcriptase (Superscript II, GIBCO BRL). The mixture was incubated first at 25 C for 10 min and then 42 C for 60 min and the reaction was terminated by heating at 95 C for 5 min. PCR amplifications were carried out using 5 μl aliquot of each RT products (cDNA templates) in 50 μl PCR reaction mixture, containing 0.2 mM dNTP, 10 mM Tris-Cl, 10 mM KCl (pH 8.3), 2.5 mM MgCl_2_, 2.5 units of Tag DNA polymerase Stoffel fragment (ApliTag, Perkin Elmer) and 50 pmol of each primer for 30 cycles. Each PCR cycle consisted of 94 C for 40 sec, 60 C for 40 sec s and 72 C for 40 sec, following an initial denaturation at 94 C for 3 min. Specific primers based on the cDNA sequences of rat ERα and ERβ were: ERα (287 bp): sense 5'-TCC TCC TAG ACC CTT CAG TGA AGC C-3'; antisense 5'-ACA TGT CAA AGA TCT CCA CCA TGC C-3'; ERβ (203 bp): sense 5'-AAA GCC AAG AGA AAC GGT GGG CAT-3'; antisense 5'-GCC AAT CAT GTG CAC CAG TTC CTT-3' and G3PDH, sense 5'-GCC ATC AAT GAC CCC TTC ATT GAC C-3'; antisense 5'-CAT CAC GCC ACA GTT TCC CGG AG-3'). The PCR products were analyzed in TBE 1.5% agarose gel and confirmed by DNA sequencing. The PCR primers used have been previously described [38,39].

### Assessment of the effects of caspase and calpain inhibitors

Cultures were treated with glutamate in the presence or absence of various amounts of calpain specific inhibitor PD150606 (selective calpain antagonist to block the calcium binding site of calpain, 1–100 μM) [40]; ALLN (calpain I inhibitor, 1–50 μM), the specific caspase-3 inhibitor (z-DEVD-fmk, 1–100 μM) and a pancaspase inhibitor (z-VAD-fmk, 1–100 μM). All protease inhibitors were prepared as 20 mM stocks in DMSO and stored at -80 C until needed.

### Determination of DNA fragmentation by gel electrophoresis

Apoptotic cells produce characteristic DNA ladders and their formation was assayed as previously described [6]. Briefly, cultured cells were washed with ice cold TBS (20 mM Tris-HCI, pH 7.6, 137 mM NaCI), DNA was isolated and 5 μg of DNA was electrophoresed on a 1.5% agarose gel for 1.5 hour at 200 V. The DNA fragments were visualized by UV transillumination after being staining with ethidium bromide.

### Measurement of AIF translocation

The cells were washed twice with ice-cold PBS, pH 7.4 and then suspended in cold isotonic buffer (250 mM sucrose, 20 mM Hepes-KOH, 10 mM KCl, 1.5 mM MgCl_2_, 1 mM Na-EDTA, 1 m M Na-EGTA, 1 mM dithiothretiol and protease inhibitors). After a 15 minute incubation on ice, cells were homogenized using a glass homogenizer on ice (25 strokes). Cell homogenates were spun at 750 g for 10 min and the pellet containing nuclei was stored at -80 C until analyzed. The supernatant was further centrifuged at 10,000 g for 15 minutes. The pellets containing mitochondria were stored at -80 C until processed. The supernatant was further centrifuged at 100,000 g for 1 hour at 4 C to obtain the cytosolic fraction (S-100). The intactness of the mitochondrial membranes was checked by assaying the mitochondrial specific enzyme glutamate dehydrogenase (GDH) in mitochondria, mitochondria treated with Triton X-100 and the cytosol (S-100) as described previously [6,15]. GDH was only detectable in mitochondria and mitochondria treated with Triton X-100, but was undetectable in the cytosol. Thus, the cytosolic fraction was free of mitochondrial contamination. Following analysis of protein concentration, the levels of AIF in those fractions were analyzed by Western blots using goat or rabbit anti-AIF antibody according as described above.

### Statistics

Each experiment was repeated at least three times and the combined data were evaluated by analysis of variance (ANOVA). Values are given as mean ± SE. Differences were considered significant at *p *< 0.05.

## Authors' contributions

***YMZ ***is a postdoctoral fellow who participated in the development of the hypothesis, study design and carried out most of the experimental work and preparation of the manuscript.

***BB ***conceived the study and participated in the development of the hypothesis, the study design, and overall direction of the study and preparation of the manuscript.
